# Curcumin and Resveratrol as Promising Natural Remedies with Nanomedicine Approach for the Effective Treatment of Triple Negative Breast Cancer

**DOI:** 10.1155/2016/9750785

**Published:** 2016-05-08

**Authors:** Amol Shindikar, Akshita Singh, Malcolm Nobre, Saurabh Kirolikar

**Affiliations:** Translational Research Laboratory, Tata Memorial Centre, Advanced Centre for Treatment, Research and Education in Cancer (ACTREC), Navi Mumbai 410210, India

## Abstract

Researchers have made considerable progress in last few decades in understanding mechanisms underlying pathogenesis of breast cancer, its phenotypes, its molecular and genetic changes, its physiology, and its prognosis. This has allowed us to identify specific targets and design appropriate chemical entities for effective treatment of most breast cancer phenotypes, resulting in increased patient survivability. Unfortunately, these strategies have been largely ineffective in the treatment of triple negative breast cancer (TNBC). Hormonal receptors lacking render the conventional breast cancer drugs redundant, forcing scientists to identify novel targets for treatment of TNBC. Two natural compounds, curcumin and resveratrol, have been widely reported to have anticancer properties.* In vitro* and* in vivo* studies show promising results, though their effectiveness in clinical settings has been less than satisfactory, owing to their feeble pharmacokinetics. Here we discuss these naturally occurring compounds, their mechanism as anticancer agents, their shortcomings in translational research, and possible methodology to improve their pharmacokinetics/pharmacodynamics with advanced drug delivery systems.

## 1. Introduction

Recent advances in the field of oncology have failed to undermine the relentless onslaught of the breast cancer epidemic. With breast cancer accounting for nearly 25% of annual global burden [1] of cancer, out of which 6–10% are metastatic at present with systemic recurrences in up to 30% of even early breast cancers [2, 3], the need of the hour is novel treatment strategies. Ground breaking work by Perou et al. [4] and Sørlie et al. [5] on the molecular classification of breast cancer in to distinct subtypes, luminal, HER2 enriched, and basal-like, confirmed the heterogeneity of this disease.

The molecular overlap between basal-like breast cancer and TNBC is close to 80%. TNBC accounts for 15–20% of all breast cancers [6] and is characterized by the lack of hormone receptor expression as well as absence of overexpression/gene amplification of HER2. TNBCs are characterized by poorly differentiated tumors with higher histological grades and high mitotic/proliferative indices. These cancers metastasize early to visceral organs most commonly the brain and lungs resulting in poor disease-free and overall survival [7]. Certain other distinctive features of TNBC include clustering of cases in premenopausal women with high BMI as well as women of African descent [8, 9] and a considerable overlap between BRCA-1 associated cancers and the triple negative phenotype [10–12]. What makes this clinical entity particularly challenging is the lack of targeted treatment in view of ER/PR/HER2 negativity thus rendering hormonal or anti-HER2 therapy redundant.

Several classes of drugs like VEGF inhibitors, tyrosine kinase inhibitors, and PARP inhibitors have been used either in the adjuvant or in the metastatic setting for TNBCs; however the results have till date been consistently disappointing with either failure to progress to phase II trials or failure to replicate phase I/II results in large phase III clinical trials.

Alternatively, natural compounds and their derivatives have been studied as dietary supplements for prevention of cancer or as new chemical entities to treat cancer [13–15] with resveratrol and curcumin as widely studied natural plant derived compounds for treatment of TNBC [15–17]. There are conflicting evidences on the role of resveratrol in prevention/treatment of TNBC. This disparity is usually observed between* in vitro* and* in vivo* studies, wherein all* in vitro* studies in the last two decades demonstrate antitumor properties of resveratrol in TNBC cell lines, while some, not all, of the* in vivo* studies do not corroborate with the* in vitro* results [15]. This disparity has been attributed to various factors in* in vivo* studies including bioavailability, mode of administration, and efficacy [18]. It is in the light of the abovementioned facts that we will review the two natural plant products and the possibility of using advanced drug delivery systems to offset their limitations as prospective therapies.

## 2. Physicochemical, Biological, and Structural Similarities of Resveratrol and Curcumin

Curcumin and resveratrol share similar biosynthesis pathways in spite of different biological origin. Source of origin is 4-hydroxycinnamic acid of the shikimate pathway. It is the most commonly employed pathway by plants to synthesize amino acids as well as aromatic/phenolic secondary compounds [19].

**Figure 1:**
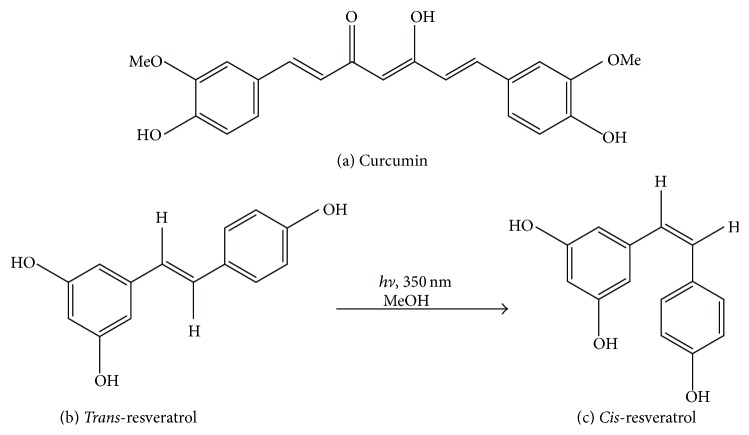
Structures of (a) curcumin and (b)* Trans-* and* Cis*-resveratrol.

Both compounds exhibit similarity in features considering their molecular topography which supports the hypothesis that the targets for absorption or efflux may be shared between them and replace each other's uptake when used in combination. With reference to molecular structures, curcumin and resveratrol show close similarities with presence of several phenolic groups as well as unsaturated carbon chains attached to hydroxyl groups [20–22] (Figure 1).

## 3. Curcumin

Curcumin (diferuloymethane) is an extract of the rhizome of turmeric (*Curcuma longa* Linn) an Indian traditional medicine [23], exhibiting antiangiogenic, antiproliferative, antitumorigenic, antioxidant, and anti-inflammatory properties in both* in vitro* and* in vivo *studies [24]. Curcumin negatively regulates various growth factors, protein kinases, transcription factors, inflammatory cytokines, cell receptors, and other oncogenic proteins. Induction of apoptosis and/or arresting different phases of the cell cycle contribute to the antiproliferative effects of curcumin in cancer cells [25, 26]. Though the antiproliferative effects of curcumin in human breast cancer cell lines, including ER positive, ER negative, and multidrug resistant cells, are time- and dose-dependent and correlate with curcumin's inhibition of ornithine decarboxylase activity [27], the mechanism of action of curcumin is largely unknown. In a study by Lv et al., human breast cancer cell lines: MDA-MB-231 (TNBC, basal-like) and MCF-7 (ER+, luminal A) treated with curcumin exhibited antitumor effects by inducing apoptosis [28]. Similarly in another study performed by Sun et al., curcumin inhibited the proliferation of MDA-MB-231 cells with the authors hypothesizing that curcumin acts via the EGFR pathway [29]. Fatty acid-binding protein 5 (FABP5), a possible prognostic marker that negates the effects of retinoic acid (RA) via the FABP5/PPAR *β*/*δ* pathway, was shown to be inhibited by curcumin thus sensitizing the RA-resistant TNBC cells to RA mediated growth suppression [30].

Investigation of curcumin as a safe remedy in phase I clinical trial was carried out at doses as high as 12 g/day but its poor systemic uptake, feeble pharmacokinetics, and rapid multiple biotransformations have made it an unfavourable chemical entity in its free form [31–33]. The use of various food ingredient formulators has also been employed to enhance the absorption and bioreactivity of curcumin [34]. Majumdar et al. have also suggested the use of a chemically stable form of curcumin albeit a more toxic analog to treat nongastrointestinal cancers. The authors also suggest that curcumin can be formulated such that it is administered intravenously instead of orally. The synergistic effects of curcumin and resveratrol have been found to inhibit colon cancer, suggesting that resveratrol also known to possess anticancer properties may act as a stabilizing agent for curcumin [35].

## 4. Resveratrol

Resveratrol is a naturally occurring polyphenol that has been reported as a cardioprotective, neuroprotective, chemopreventive agent, along with antiageing properties. Studies have been carried out using* in vitro* and animal models for studying the action of resveratrol on cancer cells and cancer related pathways. Innumerable* in vitro* studies exist in the literature on the action of resveratrol in various types of cancers like breast cancer [36–38], skin cancer [39, 40], fibrosarcoma [41], lung cancer [42, 43], gastric and colorectal cancer [44], prostate cancer [45, 46], pancreatic cancer [47, 48], hepatoma [49, 50], neuroblastoma [51], and leukemia [52, 53]. However, replication of these results in successful clinical trials has been hampered by its short half-life, poor water solubility, chemical instability, and low bioavailability when taken orally [54–56] and the overriding challenge in using resveratrol in the clinic is to achieve adequate bioavailability at tolerable dose. Drug delivery systems which direct the drugs to specific sites in the body by linking particulate systems or macromolecular carriers to monoclonal antibodies or cell specific ligands are one of the probable methods to enhance the delivery of resveratrol to achieve a therapeutic range [57].

Resveratrol acts in all three stages, that is, initiation, promotion, and progression, which affects the overall process of carcinogenesis. It promotes the cancer cells to undergo apoptosis mediated by Fas/Fas ligand, cyclin-dependent kinases cdk 1 and 2, p53, and cyclins A and B1 [58]. It arrests the cell cycle as a result of irreparable cell DNA damage of cancerous cell [59]. Resveratrol also exerts antiangiogenic property and inhibits the matrix metalloproteinases enzymes, which catalyzes the process of cancerous invasion into deeper tissues [60]. Resveratrol is involved in transcription factor NF-*κ*B modulation [61], as well as cytochrome P450 isoenzyme CYP1A1 inhibition [62]. It has also been reported that it produces synergistic activity when given in combination with anthracycline derivatives such as doxorubicine, while treating various types of cancers [63].

## 5. Bioavailability and Pharmacokinetics of Curcumin and Resveratrol as Polyphenols

Bioavailability is the rate and extent of absorption of drug at the site of action. When curcumin and resveratrol are given orally, they exhibit poor bioavailability because of short half-life and rapid elimination. To increase high intracellular uptake, large doses need to be administered, which reduces their use as supplements [64–66].

Absorption of most polyphenols does not take place in their native form [67]. These compounds undergo hydrolysis either by colonic microflora or by intestinal enzymes before their absorption. Polyphenols undergo extensive modifications during absorption process; they undergo conjugation process in intestinal cells followed by glucuronidation, methylation, and/or sulfation in the liver [68, 69]. The chemical structure rather than the concentration of polyphenols determines their bioavailability and nature of metabolite circulating in plasma. Therapeutic potency of polyphenols differs from one to another. After the process of metabolism, polyphenols produce different metabolites compared to their parental forms and circulate in the blood as well as getting absorbed in the tissues. Evaluation of potency of every metabolites is difficult [70].

### 5.1. Curcumin

Metabolism of large part of curcumin in rats via oral route was reported to be the first biodistribution study [71]. It was shown that metabolism of curcumin occurs mainly in liver [71–73]. Glucuronides of tetrahydrocurcumin (THC) and hexahydrocurcumin (HHC) were investigated as the major metabolites of curcumin in rats by Holder et al. Dihydroferulic acid along with ferulic acid in traces was considered as minor metabolite [74]. Urine of the rats treated with curcumin also showed the presence of sulfate conjugates in addition to glucuronides [75]. Findings of Pan et al. reported that 99% of curcumin conjugates present in plasma were glucuronides because of glucuronidase catalyzed hydrolysis. The same study finally concluded that curcumin produces major metabolites as tetrahydrocurcumin- (THC-) glucuronoside, dihydrocurcumin–glucuronoside* in vivo* [76].

In another study conducted on healthy humans, pharmacokinetics of a curcumin preparation in healthy human volunteers was examined at 0.25 to 72 hr after a single oral dose. Given doses of curcumin were 10 g (*n* = 6) and 12 g (*n* = 6). When serum sample of subjects was analyzed using HPLC (50 ng/mL as limit of detection) as an analytical technique, free curcumin was detected in one subject only at any of the 14 time points. However, samples of all subjects were detected with the presence of curcumin glucuronides and sulfates. Plasma samples were not detected with free curcumin from any other subject [77].

### 5.2. Resveratrol

Resveratrol (3,4′,5-trihydroxy-*trans*-stilbene) is a stilbene class based polyphenolic compound. Rapid and extensive metabolism of the resveratrol and formation of several different metabolites as resveratrol glucuronides and resveratrol sulfates consequently result in zero bioavailability, when administered orally [78].* In vitro* experiments involved treatment and incubation of human hepatocytes, human liver microsomes, and rat hepatocytes. Rats and mice were administered with resveratrol via oral and intraperitoneal routes* in vivo* studies. When samples of rat urine, mouse serum, human hepatocytes, rat hepatocytes, and human liver microsomes were analyzed by HPLC for resveratrol metabolites using methanolic extracts,* trans*-resveratrol-3-*O*-glucuronide and* trans*-resveratrol-3-sulfate were detected abundantly in all samples. Structures of these conjugates were confirmed on incubation with beta-glucuronidase and sulfatase which releases free resveratrol [79].

In another study, 500 mg of resveratrol immediate-release uncoated caplets was administered to ten subjects. Initially, a starting dose of 1 g was given followed by sequentially increasing it to 2.5 g and 5.0 g. After obtaining pharmacokinetic data at 5 g dose, 10 subjects were given dose of 0.5 g.

Plasma and urine samples showed the presence of two monosulfates, one disulfate, two monoglucuronides, and one glucuronide-sulfate when subjected to HPLC analysis. These findings reconfirm the avid metabolism of resveratrol in humans.

Short half-life, nonretention ability, rapid elimination, and undesirable degradation/biotransformation lead to the low bioavailability of parent molecule of curcumin and resveratrol at the site of action [80].

## 6. Tumour Vasculatures and Enhanced Permeability and Retention (EPR) Effect and Its Impact on Advanced Drug Delivery Systems (ADDS)

Over a period of decade, many types of cancers are being treated by conventional chemotherapy using small molecules. Lack of tumour selectivity results in developing severe adverse side effects; consequently drug doses need to be used in less volumes. Drug efficacy also remains suboptimal [81]. To improve the bioavailability and therapeutic efficacy of hydrophobic drugs, development of site specific tumour targeted chemotherapy is the most effective approach for treating different types of cancers most successfully with ease.

Tumour specific targeting at vascular and tissue level can be achieved by the breakthrough discovery of enhanced permeability and retention (EPR) effect of solid tumours. Pathophysiological and anatomical features of tumor vessels are attributed to the rate and extent of EPR effect in most solid tumours. As a result, many researchers across the globe have been employing the concept of tumor targeting drug delivery following the concept of EPR effect to develop efficient and effective anticancer drugs [82].

Tissues undergoing some pathological conditions result in diseases such as cancer or inflammation exhibits the characteristic abnormal vasculature, which enhances the permeability, retention, and extravasation of macromolecular drugs. Activation of vascular permeability factors as well as deregulated angiogenesis contributes to EPR effect of solid tumours. Important characteristic features of abnormal vasculature include formation of large fenestrations (300 nm to 4,700 nm in size) between the endothelial cells because of its discontinuous features. Activation of proangiogenic and antiangiogenic molecules and imbalance in their expression lead to the formation of large fenestration [83]. Abnormal porous vasculature, compression of the lymphatic vessels as a result of increase in number of cancer cells, and dysfunctional lymphangiogenesis, which causes lymphatic drainage impairment in tumor tissues, are also among other factors.

**Figure 2:**
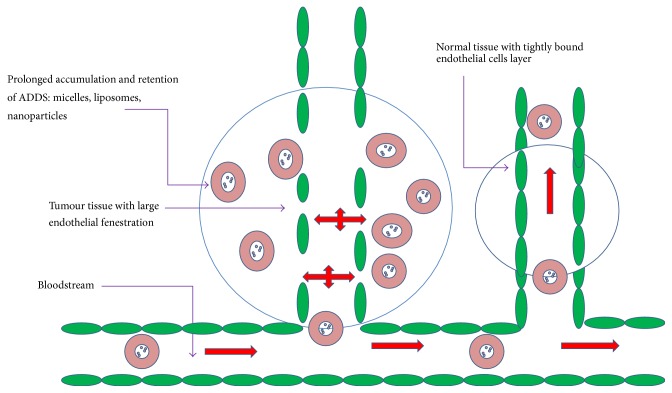
Schematic representation of enhanced permeability and retention effect: passive targeting by ADDS (macromolecular extravasation).

Interstitial fluid of tumours, which constitutes any constructs or macromolecules including encapsulated drugs or antibodies, is retained for longer period than in normal tissues [84–87] (Figure 2). Healthy tissues do not exhibit EPR effect due to normal vasculature with functional lymphatic drainage (Figure 2).

Favourable alteration of drug pharmacokinetics can be achieved by the ability of macromolecular formulations to extravasate and penetrate the tumours. Encapsulation of drugs protects them from undergoing undesirable metabolism/biotransformation and degradation, which results in plasma half-life prolongation and retention. Macromolecular drugs exhibit different biodistribution pattern compared to free drugs. Macromolecules penetrate the tissues and enter the tumour through the process of endocytosis. A study has supported the hypothesis of EPR effect wherein a liposomal formulation of doxorubicin was administered and doxorubicin induced cardiotoxicity was found to be decreased in the patients [88]. Based on these facts and evidences, it can be considered that advantage of abnormal vasculature may contribute to achieving EPR effect for naturally occurring polyphenols including resveratrol and curcumin as well as other highly hydrophobic, insoluble, unstable, less bioavailable compounds using various compatible ADDS methods.

## 7. Advanced Drug Delivery Systems

To overcome the problems of poor bioavailability and poor pharmacokinetics associated with curcumin and resveratrol, numerous ADDS systems like adjuvants, nanoparticles, liposomes, micelles, phospholipid complexes, dendrimers, nanoemulsions, nanogels, and nanogold are being developed by performing extensive studies.

### 7.1. Adjuvants

#### 7.1.1. Curcumin

Adjuvants are known to have an inhibitory activity on hepatic and intestinal glucuronidation. Use of piperine as an adjuvant with curcumin was administered in rats and healthy human volunteers. 2 g/kg of curcumin alone showed a maximum serum curcumin level of 1.35 (0.23 *μ*g/mL at 0.83 h), whereas concomitant administration of piperine (20 mg/kg) increased the serum concentration of curcumin for a short period of time with a significant increase in its maximum peak level. A decrease in the elimination half-life and clearance of curcumin resulted in an increase of bioavailability in rats. However, in human volunteers consuming a dose of 2 g curcumin alone, serum levels were either not detectable or found to be very low and piperine produced a 2000% increase in bioavailability when administered concomitantly [89].

#### 7.1.2. Resveratrol

It has been known that “*β*-glucan” has been used as an adjuvant, drug carrier, or in combination with a drug or compound such as resveratrol by developing drug delivery system to enhance the bioavailability. A study was performed by a laboratory to know the possible combination/synergistic effects of *β*-glucan and resveratrol on immune reactions. Significant synergetic effects were reported in all cases, wherein potency of these compounds on some genes expressions (such as NF-*κ*B2, Cdc42, and Bcl-2) in breast cancer cells was tested. Cdc42 levels were found to be upregulated only when resveratrol and glucans were used in combination [90].

### 7.2. Nanotechnology (Nanoparticles)

**Figure 3:**
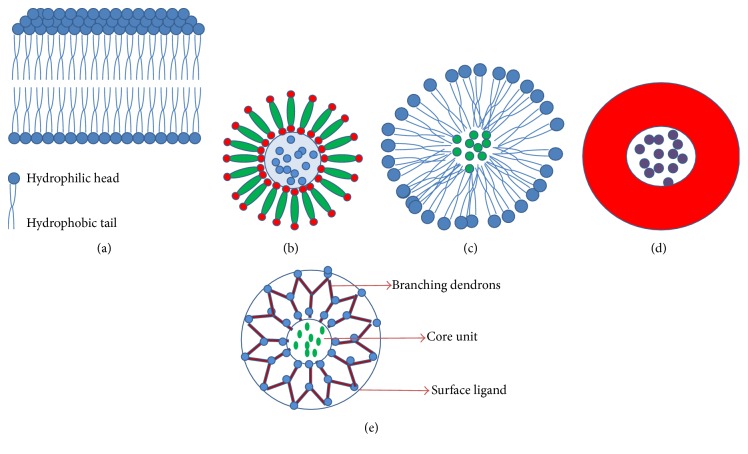
Structures of (a) liposome bilayer, (b) liposome, (c) micelle, (d) polymeric nanoparticles, and (e) dendrimer.

Nanoparticle technology is a promising drug delivery system developed to enhance the bioavailability of many therapeutic drugs especially highly hydrophobic agents like curcumin (Figure 3(d)).

#### 7.2.1. Curcumin

Limited studies have shown the application of curcumin nanoparticles. Bisht et al. reported the synthesis, physicochemical properties, and cancer related application of “nanocurcumin” (size less than 100 nm), a polymer-based nanoparticle of curcumin. In a study it was reported that nanocurcumin showed similar* in vitro* activity as that of free curcumin in pancreatic cell lines. Nanocurcumin also inhibited activation of the transcription factor NF-*κ*B and reduced steady state levels of proinflammatory cytokines like interleukins and TNF-R similar to free curcumin [91].

Solid lipid nanoparticles (SLNs) which are known as lipid-based drug delivery have become an area of focus in recent times. SLNs as the name suggests are produced by using lipids that are in solid phase at room and body temperatures and are preferred because of their physical stability and ability to protect labile drugs from enzymatic and chemical degradation and controlled release. Curcuminoid having a size of 450 nm loaded solid lipid nanoparticles (SLNs) was found to be stable for 6 months at room temperature and gave a prolonged and sustained* in vitro* release of curcuminoids for 12 hrs. Additionally, light and oxygen sensitivity of curcuminoids were found to be significantly reduced by formulating the curcuminoids into nanoparticles [92].

#### 7.2.2. Resveratrol

The use of resveratrol in combination with serum albumin in a nanoformulation has been found to significantly inhibit the growth rate of human primary ovarian cancer cells as compared to free resveratrol when implanted subcutaneously [93]. Shao et al. used mPEG poly(epsiloncaprolactone)-based nanoparticles incorporating resveratrol and demonstrated a significantly higher rate of cell death as compared to an equivalent dose of free resveratrol in glioma cells [94]. Incorporating resveratrol in SLN results in decreasing cell proliferation which has been shown to be beneficial in preventing skin cancer [95]. In another study, resveratrol NPs uptake by PCa cell lines was found to be high. In addition, when the PCa, DU-145, and LNCaP were treated with free resveratrol and nanoresveratrol all three cell lines showed significantly elevated cytotoxicity compared to that of free resveratrol at different concentrations (from 10 *μ*M to 40 *μ*M). It proves the consistent sensitivity of nano-RSV towards both the hormone-sensitive LNCaP cells and androgen-independent DU-145 prostate cancer cell lines [96].

### 7.3. Nanosponges

Nanosponges are nonmutagenic, nonallergenic, and nontoxic and have been used to transport and deliver anticancer drugs.

#### 7.3.1. Curcumin

In a study, researchers used cyclodextrin-based nanosponges to enhance curcumin's solubility. They employed dimethyl carbonate as a cross-linker and formulated the complex of *β*-cyclodextrin-curcumin nanosponge. The solubilization efficiency of loaded nanosponges was found to be more compared to free curcumin and *β*-cyclodextrin complex. Interactions of curcumin with nanosponges were confirmed by the characterization of curcumin nanosponge complex. Also, drug release of curcumin in* in vitro* studies was well controlled over a prolonged time period and the complex was found to be nonhemolytic [97].

#### 7.3.2. Resveratrol

Cross-linking of different types of cyclodextrin (CD) with cross-linker compound such as carbonyldiimidazole produces nanosponges. They exhibit high solubilization property for the molecules which are poorly soluble in nature. Nanosponges are spherically shaped solid particles [98].

In a study, nanosponges of resveratrol were synthesized. It significantly enhanced the stability as well as solubility of the molecule. Drug permeability was enhanced in* in vitro* studies on porcine skin and* in vivo* rabbit buccal mucosa using resveratrol-loaded nanosponges [99].

In another study, nanosponges are nonmutagenic, nonallergenic, and nontoxic and have been used to transport and deliver anticancer drugs [100]. Nanosponges prepared from hyper-cross-linked *β*-cyclodextrins have been used as a carrier in* in vitro* studies with tamoxifen on MCF-7 cells [101]. Similarly William et al. [102] prepared nanosponges from poly(valerolactone-allylvalerolactone) and poly(valerolactone-allylvalerolactone-oxepanedione) and used temozolamide in a drug release study to treat brain tumors* in vivo *and* in vitro*. Nanosponges of *β*-cyclodextrins carrying paclitaxel were tested for bioavailability and cytotoxicity* in vivo* in Sprague Dawley rats. As compared to control group, orally administered paclitaxel loaded PLN group showed 3-fold increase in area under the plasma concentration time curve which was found to be significant (*p* < 0.05).

Considering the results obtained from the study, it is evident that the oral bioavailability of paclitaxel can be enhanced using PLN as a most promising new formulation while avoiding the use of cremophor El: Ethanol in Taxol. Similarly, in an* in vitro* study, when MCF-7 cells were treated with paclitaxel nanosponge complex to determine cytotoxic efficacy, it was observed that cytotoxicity of paclitaxel nanosponge complex was reported to be higher against this cell line as compared to the paclitaxel group [103, 104].

### 7.4. Liposomes

Liposomes carry both hydrophilic and hydrophobic molecules and are known as excellent drug delivery systems (Figures 3(a) and 3(b)).

#### 7.4.1. Curcumin

*In vitro* and* in vivo* antitumor activity against human pancreatic carcinoma cells using liposomal curcumin demonstrated that liposomal curcumin suppressed the pancreatic carcinoma growth in xenograft models by inhibiting tumor angiogenesis [105].

In another study,* in vitro* and* in vivo* effects of liposomal curcumin on proliferation, apoptosis, signaling, and angiogenesis in human pancreatic carcinoma cells were studied. Results of the study showed NFk-B downregulation, growth suppression, and apoptosis induction* in vitro*.* In vivo* results reported antitumor and antiangiogenesis activity as well [106].

#### 7.4.2. Resveratrol

In one study, when mitochondrial targeting resveratrol liposomes were used, this induced apoptosis in both nonresistant and resistant cancer cells by dissipating mitochondrial membrane potential. It also increased caspase-9 and caspase-3 activities. Significant antitumor efficacy was exerted by resveratrol liposomes in in xenografted resistant A549/cDDP cancers in nude mice and tumour spheroids by deep penetration [107].

In another study, viability of HEK 293 cells and their photoprotection after UV-B irradiation was tested with free and liposomal resveratrol. Interestingly, cell viability was found to be decreased at 100 *μ*M concentration and cell proliferation increased at 10 *μ*M and achieved the most effective photoprotection. This study showed effectiveness of resveratrol at 10 *μ*M and also toxicity at higher concentrations considering the changes in apoptotic features and cell shape and its detachment [108].

### 7.5. Polymeric Micelles and Phospholipids Complexes

Micelles and phospholipid complexes can improve the gastrointestinal absorption of natural compounds by decreasing undesirable rapid metabolism and early elimination resulting in higher plasma levels and improved bioavailability (Figure 3(c)).

#### 7.5.1. Curcumin

*In vitro* model of everted rat intestinal sacs, intestinal absorption of curcumin, and curcumin's micelle with phospholipid as well as bile salt was investigated.* In vitro* intestinal absorption of curcumin increased from 47% to 56% in case of micellar curcumin formulation [109]. Polymeric micellar curcumin pharmacokinetic study showed increase in biological half-life to 60-fold for curcumin in rats compared to curcumin solubilized in a mixture of DMA, PEG, and dextrose [110]. In one study, curcumin (100 mg/kg) and curcumin–phospholipid complex (corresponding to 100 mg/kg of curcumin) was administered to Sprague–Dawley male rats orally. Maximum plasma curcumin level of 600 ng/mL, 2.33 hours, was detected after oral administration of curcumin–phospholipid complex as opposed to that of free curcumin having maximum plasma concentration of 267 ng/mL after 1.62 hours of oral dosing with a 1.5-fold increase in the half-life of curcumin [111].

#### 7.5.2. Resveratrol

In a study carried out by Narayanan et al. in which a combination of resveratrol and curcumin was used, a significant decrease of prostatic adenocarcinoma in PTEN knockout mice and* in vitro* studies on PTEN-CaP8 cancer cells revealed that resveratrol in combination with curcumin inhibited cell growth and induced apoptosis [112].

To increase the effectiveness of resveratrol, drug delivery system like nanocapsules can be used to target drugs at specific sites within the body in the field of cancer biology. The* trans*-resveratrol-loaded lipid core nanocapsule (RSV-LNC) was used to test its antiglioma activity on C6 glioma cell line* in vitro* and on brain implanted C6 cells in* in vivo* models.* In vitro* studies indicated that RSV-LNC decreased the cell viability of C6 glioma cells to a much greater extent as compared to resveratrol when used alone in solution.* In vivo* studies RSV-LNC also showed a marked decrease in the size of the tumour, suggesting that RSV-LNC nanocapsules could be used effectively in the treatment of gliomas [113].

### 7.6. Polymeric Drug Conjugates with Ligands

Ligands are one of the biomarkers that can be used to differentiate between cancer tissue and normal tissue. Attaching ligands to the surface of nanoparticles can help recognize and bind selectively to the receptors that are expressed on tumour cells. This technique will help deliver high doses of anticancer drug directed specifically to the tumour cells sparing the normal cells, thus decreasing the side effects associated with the drug. Inclusion of a targeting antibody or ligand into polymer-drug conjugates has been the most suggested approach to encounter these limitations.

To support this hypothesis, researchers in a study produced a formulation comprising heparin as a carrier and a new folate receptor-targeted paclitaxel nanoparticle (heparin-folate-Taxol (paclitaxel), HFT) and evaluated its activity in nude mouse animal models. Results of this study reported that heparin-folate-Taxol (paclitaxel) showed increased potency against the tumor xenografts growth of human KB and paclitaxel-resistant KB derivatives as compared to binary heparin-Taxol or free paclitaxel [114].

In another study, as compared to free paclitaxel treated MCF-7 and MCF-7/Adr cells, transferrin-conjugated paclitaxel loaded (poly(lactic-co-glycolic acid) polymer) nanoparticles showed elevated inhibitory effects on growth of the same cells [115]. In one more study, liposomes conjugated transferrin increased the transfection efficacy of p53, which lead to the ionizing radiation causing sensitization of the transfected cancer cells/xenografts [116]. These studies were performed on the hypothesis based on transferrin as a target for tumor specific drug delivery because it was already investigated that tumor tissues overexpress transferrin receptors as compared to normal tissues [117].

### 7.7. Dendrimers

In a study, PAMAM encapsulated curcumin and free curcumin were tested on T47D breast cancer cell line for their comparative antiproliferative effect. Using TRAP assay, telomerase activity was studied after 24 hrs of incubation. Inhibitory effect was found to be increased in telomerase activity. No cytotoxicity on cancer cells was found when treated with PAMAM dendrimers encapsulating curcumin. It also showed increase in antiproliferative activity of curcumin [118]. (General representation of dendrimer is as shown in Figure 3(e)).

In another study, several cancer cell lines treated with Curc-OEG inhibition were reported at high level due to apoptosis. Reduction in tumor weights and tumor numbers was also observed when Curc-OEG was intravenously injected in the SKOV-3 tumors xenografted athymic mice as well as subcutaneous (mammary fat pad) MDA-MB-468 tumors. Also no acute and subchronic toxicities in mouse visceral organs at high doses were investigated due to Curc-OEG. Doxorubicin and camptothecin anticancer drugs can be carried by Curc-OEG nanoparticles as drug carriers to enhance the cytotoxicity in drug resistance cancer cells successfully [119].

### 7.8. Nanoemulsions

As discussed above, curcumin and resveratrol polyphenolic compounds are lipophilic in nature and have very low solubility in water. As a result of poor solubility, they show lower absorption capacity in the gastrointestinal tract. This leads to limited bioavailability. An effective approach could be nanoencapsulation in o/w nanoemulsions-based delivery systems for incorporating bioactive compounds resveratrol and curcumin.

The lipid droplets are nanometric in size (50–200 nm) dispersed in hydrophilic phase using a suitable emulsifying agent at the oil/water interface which is known as “nanoemulsions” [120]. A method used as energy-intensive comminution process, using high pressure homogenization, produces a nanoemulsion [121]. Enhancement of passive transport mechanism which is related to concentration gradient across the cell membrane by subcellular size of the nanocapsules leads to improving absorption and bioavailability of resveratrol and curcumin [122]. Authors are conducting several studies in the field of nanocapsulation, extensively on two most promising polyphenols, that is, curcumin [123] and resveratrol, which are known for their many beneficial effects on the human health (anticancer, anti-inflammatory, antimicrobial, antioxidant, and chemopreventive activity), However they face many challenges due to poor bioavailability which limits their clinical use [124]. This study focused on the fabricated stable nanoemulsions using soy lecithin, sugar ester, and modified starch as natural and food acceptable ingredients to encapsulate two polyphenolic compounds, curcumin and resveratrol. This approach is followed for improving their dispersibility in aqueous systems.

At the end of this study, it was observed that nanoemulsion-based delivery systems by encapsulation of polyphenols improved their water dispersibility and protected them from degradation as well as preserving the antioxidant activity. It was also observed that stability of resveratrol was improved when resveratrol (0.01% wt) was encapsulated in peanut oil-based nanoemulsions as shown by the significant reduction of the chemical degradation of* trans*-resveratrol to* Cis-*resveratrol. As far as curcumin (0.1% wt) is concerned, it was encapsulated in solid lipid nanoemulsions that trapped the compound in a solid matrix, which lead to improved solubility in aqueous systems and to avoiding the recrystallization and settling of the bioactive compound over time [125].

### 7.9. Nanogels

Nanogels are crosslinked polymer network ranging size between 10 and 200 nm.* In vitro* studies were performed on breast cancer, melanoma, and pancreatic cell lines. Cell lines were treated by nanoparticles conjugated curcumin formulation. Results were found to be very interesting. Nanocurcumin increased stability of curcumin, enhanced fluorescence effects, developed bioavailability, improved anticancer effects, got better controlled release, prolonged half-life, and enhanced treatment of melanoma [126, 127].

## 8. Evidence for Combining Resveratrol and Curcumin

While evidence for combining resveratrol and curcumin in the treatment of triple negative breast cancer is lacking,* in vitro* studies have demonstrated synergistic antiproliferative/apoptotic effects in colon [29] and hepatocellular carcinoma [128]. Additionally resveratrol has been shown to enhance both* in vivo* and* in vitro* antitumoral effects of curcumin in head and neck carcinoma by increasing the cleavage of PARP-1 and the Bax/Bcl-2 ratio, by inhibiting the phosphorylation of ERK1 and ERK2, and the expression of LC3 II simultaneously with the formation of autophagic vacuoles [129]. More recently resveratrol and curcumin synergistically caused apoptosis in cigarette smoke condensate transformed breast cancer epithelial cells, via p21 mediated inhibition of the Hedgehog-Gli cascade signaling pathway [130]. Coencapsulation of resveratrol and curcumin in lipid core nanocapsules [131] and polymeric micellar codelivery of resveratrol and curcumin [132] have been shown to improve their antioxidant effects and decrease* in vitro* doxorubicin induced cardiotoxicity, respectively. Despite the lack of more robust evidence for using these two compounds in combination, it would be worthwhile to explore this in future studies.

In one study, wherein resveratrol, curcumin, and quercetin were used as combination therapy (200 nm in size) with or without piperine. affected an* in vitro* permeability model using apical-to-basal permeability across intact caco-2 monolayers. Quercetin, resveratrol, and curcumin were applied apically alone or in combination at 50 *μ*M and measured in the basal chamber at 30 min.

Greatest enhancement in permeability was received by resveratrol when combined with other agents: quercetin (310%), curcumin (300%), and quercetin and curcumin (323% and 350% with piperine). Increased permeability was recorded in case of curcumin when combined with quercetin alone (147%) and both quercetin and resveratrol (188%); addition of piperine resulted in a 229% increase in permeability [133].

## 9. Conclusion

Curcumin and resveratrol appear to be promising anticancer agents but poor solubility, bioavailability, pharmacokinetics, and biodistribution limit their routine use in patients. Rapid elimination, short half-life, undesirable degradation/biotransformation, and instability are the major problems associated with both polyphenols. To overcome these drawbacks, ADDS in conjunction with these drugs present an exciting, novel, and efficient alternative. Evidence for their routine use appears to be limited at present and thorough and extensive studies are mandatory prerequisites prior to testing them on humans. Hence, ADDS has become a common interest and central point for many institutional and pharmaceutical research laboratories across the globe to deliver the drug restricted to the tumour site and reduce the side effects/adverse reactions associated with it.

Considering the literature, we can herby conclude that ADDS is the most promising way to encounter the bioavailability and pharmacokinetic issues associated with naturally occurring potential polyphenolic compounds such as curcumin and resveratrol to treat TNBC in most efficient and effective manner.
